# The Montreal Veterinary Medical Association

**Published:** 1893-03

**Authors:** 


MONTREAL, CANÁDA, JAN. 12, 1893.

*The Montreal Veterinary Medical Association.*—The Montreal Veterinary Medical Association met in the college lecture room this evening with the President, Dr. Mills in the chair ; Vice-President, Dr. Baker, was also present.

Mr. Cleaves reported a case of "Nervous Prostration in the Dog," a Cocker spaniel bitch, which had come under his care. The most prominent symptoms were: animal lying on its side, unable to rise, muscles twitching, which was the first symptom noticed. Eyes clear, pulse too rapid to be counted, temperature 103 degrees, respirations hurried.

She had had puppies seven in number, three weeks previous, three of which had been disposed of; she seemed excited about the puppies. Treatment: Was removed from sight of puppies, and made comfortable. Pot. Bromide gr. x, was given every two hours, and when any pain was present Tr. Opii m x.

After the animal began to improve, at the end of eight hours, two doses of the following were given with an hour's interval :

*R.* Pot. Bromide gr. v.
Tr. Bellad. m viii.
Tr. Aconite m v.
Chloral Hydrate gr. v
Aquae q. s.

At the end of ten hours she appeared normal, except for weakness.

Dr. Baker stated that he frequently met an affection in bitches, similiar to this, after whelping. They rarely died.

Mr. Orr took for his subject Laminitis, Peditis or founder, a term supposed to be given, because the animal resembles a ship foundered at sea, being perfectly helpless.

In this paper the causes, symptoms, pathology and treatment were most thoroughly dealt with. [See page 3.]

As this is a very common disease as seen in practice, and as all the readers are more or less aquainted with nature, causes, symptoms, pathology and treatment, will pass on to the discussion which is brought out. The manner in which the writer defended his paper is worthy of comment, and showed that he understood the disease, both theoretically and practically.

The following are some of the points dwelt on in the discussion :

*Ques.* Does animal stand when all four feet are affected? *Ans.* May stand all the time or may be intermittent with lying. When he stands, the front feet are extended and hind feet advanced more under belly.

*Ques.* What is the action in blood letting? *Ans.* To lower blood pressure and relieve arterial tension.

*Ques.* Does lowering blood pressure act locally or generally? *Ans.* Affects the whole body.

*Ques.* Would you use Amile Nitrite or Nitro-glycerine to relieve Art. tension? *Ans.* Never saw it tried, or read anything concerning it in this disease.

*Ques.* Why are feet affected from overfeeding? *Ans.* The whole system is affected.

*Ques.* Can cold water produce laminitis when animal is warm?

This brought out a discussion. Dr. Gadsden, of Philadelphia, says it can not, and has experimented in this line with horses of St. R. R. of that city with negative results, also with his own horses.

*Ques.* How does Super-purgation set up laminitis? *Ans.* Through the nervous system as an irritation.

*Ques.* Is there anything contrary to local blood letting? *Ans.* It is the course to be adopted.

*Ques.* Can Metastasis be explained through a reflex? *Ans.* I believe it is not the explanation.

*Ques.* How much blood should be drawn? *Ans.* Enough to make an impression on the pulse.

Dr. Baker was called upon to say a few words. He thought it well to keep our Physiology with us in our treatment of disease.

"Metastasis" is generally a mistake in diagnosis.

May have laminitis, associated with other diseases.

Blood letting goes in waves. Have had as good results without blood letting. Generally good to bleed if animal is in first stage.

Local blood letting may prevent sloughing of the sole.

Dr. Mills thought there was a great deal in "laminitis" that suggests "gout." The structure of the horse's foot should be kept clearly in mind. Metastasis begins in the endoderm. Metastasis is to be explained through the nervous system largely, but the term is greatly abused. Life itself is a kind of reflex.

